# GeNeCK: a web server for gene network construction and visualization

**DOI:** 10.1186/s12859-018-2560-0

**Published:** 2019-01-07

**Authors:** Minzhe Zhang, Qiwei Li, Donghyeon Yu, Bo Yao, Wei Guo, Yang Xie, Guanghua Xiao

**Affiliations:** 1 Quantitative Biomedical Research Center, University of Texas Southwestern Medical Center, 5323 Harry Hines Blvd., Dallas, 75390 TX United States; 2 Department of Clinical Sciences, University of Texas Southwestern Medical Center, 5323 Harry Hines Blvd., Dallas, Texas United States; 3 Department of Statistics, Inha University, Incheon, South Korea; 4 BioHPC team, Lyda Hill Department of Bioinformatics, University of Texas Southwestern Medical Center, 5323 Harry Hines Blvd., Dallas, Texas United States; 5 Harold C. Simmons Cancer Center, University of Texas Southwestern Medical Center, 5323 Harry Hines Blvd., Dallas, 75390 Texas United States

**Keywords:** Gene network, Gene network, Statistical method, Web server, Correlation, Likelihood, Bayesian, Mutual information, Ensemble, Hub gene, Visualization

## Abstract

**Background:**

Reverse engineering approaches to infer gene regulatory networks using computational methods are of great importance to annotate gene functionality and identify hub genes. Although various statistical algorithms have been proposed, development of computational tools to integrate results from different methods and user-friendly online tools is still lagging.

**Results:**

We developed a web server that efficiently constructs gene networks from expression data. It allows the user to use ten different network construction methods (such as partial correlation-, likelihood-, Bayesian- and mutual information-based methods) and integrates the resulting networks from multiple methods. Hub gene information, if available, can be incorporated to enhance performance.

**Conclusions:**

GeNeCK is an efficient and easy-to-use web application for gene regulatory network construction. It can be accessed at http://lce.biohpc.swmed.edu/geneck.

**Electronic supplementary material:**

The online version of this article (10.1186/s12859-018-2560-0) contains supplementary material, which is available to authorized users.

## Background

A gene regulatory network (GRN) describes biological interactions among genes and provides a systematic understanding of cellular signaling and regulatory processes. It depicts how a set of genes interact with each other to form a functional module and how different gene modules are related. A typical GRN approximates a scale-free network topology with a few highly connected genes (i.e. hub genes) and many poorly connected nodes [1]. These hub genes are master regulators in a gene network, and usually play essential roles in a biological system. Investigations of GRN can facilitate the systematic functional annotation of genes [2] and help identify the hub genes, which may lead to potential clinical applications [3].

Reverse engineering approaches to construct gene networks from transcriptomic data have greatly facilitated biomedical research. Statistical methods proposed for inferring network structure can be categorized into four classes: 1) probabilistic network-based approaches, mainly Bayesian networks (BN); 2) correlation-based methods; 3) partial correlation-based methods; and 4) information theory-based methods [4]. Comparative evaluation among different methods for constructing large scale GRNs revealed the strengths and weaknesses of each method with respect to different scenarios, with no single method outperforming others universally [5]. An ensemble-based network aggregation (ENA) method was proposed to integrate different methods to improve the accuracy of network inference [6]. Recent advancements in statistical methods have extended algorithms to incorporate prior knowledge of hub genes [7]. Besides above statistical methods that aim to infer the latent covariance matrix of all the components in a graph using gene expression data, other algorithms like Petri Nets [8] and ordinary differential equations (ODE) [9] focus more on simulating the dynamics of specific pathways that involve important disease genes.

**Fig. 1:**
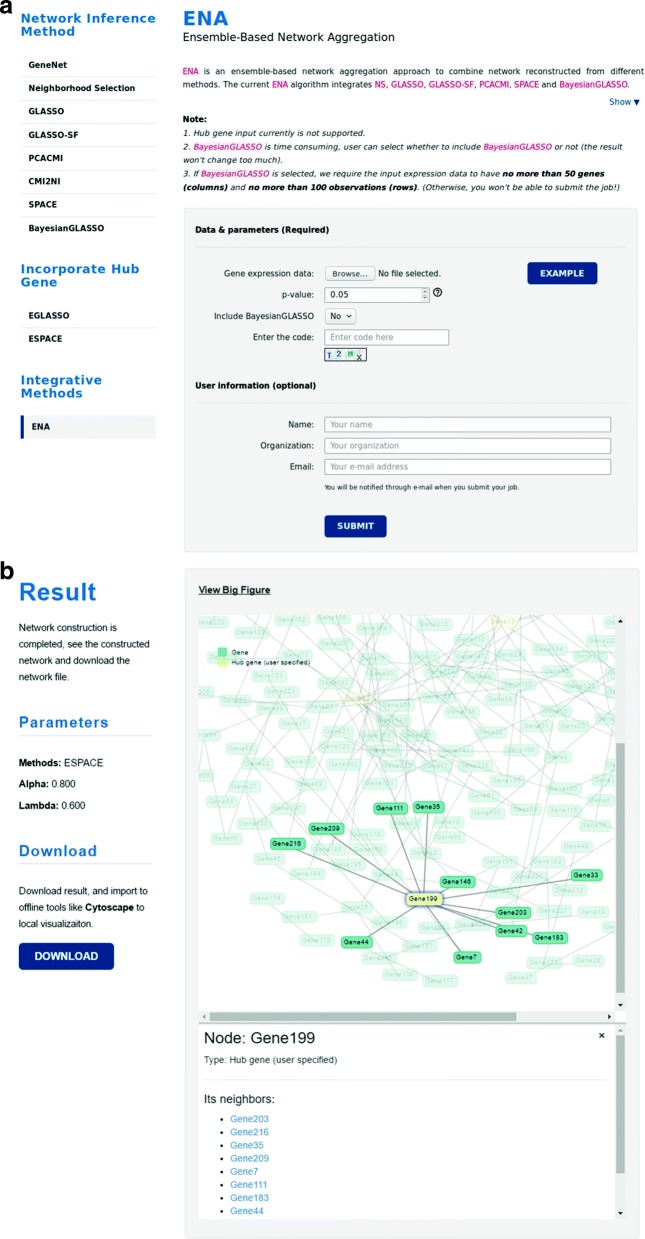
**a** Web interface of GeNeCK analysis page. **b** Visualization of constructed network in GeNeCK results page

Despite the development of various computational methods and corresponding R packages for inferring gene-gene interactions, implementation of those algorithms with graphical interface is still lagging. CoExpNetViz [10] is an online tool developed for constructing co-expression networks in plant research, but its application is limited by simple statistics and compulsory “bait” genes input. To provide easy accessibility for the network construction tool, we introduce a web server called GeNeCK (Gene Network Construction Tool Kit, see Fig. 1) which allows users to upload their own gene expression data and choose their preferred method to infer and visualize the network, as well as integrate different methods to obtain a more confident result.

## Implementation

GeNeCK is a web server (http://lce.biohpc.swmed.edu/geneck) with a user-friendly graphical interface. A quick user guide on how to upload data and submit jobs is provided on the website and in the supplementary material (Additional file 1: Figure S9). GeNeCK offers the flexibility for experienced users to select methods and set preferred parameters. Using ENA is more straightforward for most users since it generally performs well in all scenarios, does not require choosing tuning parameters, and can provide a *p*-value for each connection, which indicates the statistical significance of the connection. The constructed network will be displayed on the website once the job is finished (Fig. 1). Genes with a high degree of connection (i.e. hub genes) will be plotted with different colors. Users can interactively explore the constructed network. Clicking on a specific gene will highlight the gene itself along with its connected neighbors, and the corresponding information will be displayed at the bottom (Fig. 1).Although the current version of GeNeCK does not provide a function for users to download the figure, users can use screenshot software tools to get the figure for the network structure. We recommend that users download and import the constructed network structure into other visualization tools, such as Cytoscape, for further visualization and analysis (Additional file 2: Figure S10).

## Methods

GeNeCK allows users to construct network using 11 different methods (summarized in Additional file 3: Table S1). Readers can refer to Yu et al. [7] for a comprehensive review of the different network construction methods.

### Network inference methods

Partial correlation-based methods calculate the inverse covariance matrix ***Ω*** (also known as the precision matrix) of gene expressions, in which *ω*_*j*,*h*_=0 indicates gene *j* and *h* given the expressions of all the other genes is conditional independent. GeneNet [11] employs Moore-Penrose pseudoinverse and bootstrap methods to obtain a shrink estimate of ***Ω***. Meinshausen and Bühlmann [12] proposed the neighborhood selection (NS) method, which converts the precision matrix estimation problem to a regression problem by fitting a LASSO to each gene using others as predictors. Sparse partial correlation estimation (SPACE) is a joint spare regression model developed by Peng et al. [13], which resolves a symmetrically constrained and *L*_1_-regularizated regression problem under high-dimensional settings.

Likelihood-based approaches, such as graphical LASSO (GLASSO [14]) and GLASSO with a reweighted strategy for scale-free networks (GLASSO-SF [15]), optimize a penalized maximum likelihood function to estimate ***Ω***. Bayesian graphical LASSO (BayesianGLASSO [16]) is a fully Bayesian treatment of GLASSO that uses a double exponential prior and employs a block Gibbs sampler for exploring the posterior distribution.

Mutual information (MI) is a measure in information theory of pairwise dependency between two variables. Zhang et al. [17] proposed a path consistency algorithm based on conditional mutual information (PCACMI) to infer graphical structure, and further conditional mutual inclusive information-based network inference (CMI2NI [18]) method that improves the PCACMI method.

### Hub gene incorporation

Gene networks usually have scale-free characteristics. In other words, there are usually a few hub genes regulating many others. In practice, most of such hub genes in biological pathways have been well studied and validated through biological experiments. To properly incorporate this prior knowledge, Yu et al. [7] proposed extended sparse partial correlation estimation (ESPACE) and extended graphical LASSO (EGLASSO) methods. In these methods, during the covariance estimation of original SPACE and GLASSO methods, hub gene information can be incorporated to improve the network inferences.

### Network integration

An ensemble-based network aggregation (ENA) method [6] combines networks reconstructed from different methods. The original ENA algorithm does not report the confidence level of estimated edges. To derive the *p*-value of an edge between a pair of genes, we adapted ENA by implementing an additional permutation step to generate the distribution of null hypothesis. We first permute the given gene expression dataset to obtain a resampled dataset *D*^(*m*)^. Then we implement the ENA algorithm to get the ensemble rank matrix $\tilde {R}^{(m)} $ for this dataset. This procedure is repeated *M* times. The empirical null distribution *F*^null^ of all possible pairwise connection for *p* genes can be obtained based on all the harmonic means in the *M* permutations, i.e. $\left \{\tilde {r}^{(m)}_{jh},\,m=1,...,M,1\leq j < h \leq p\right \} $. Then the *p*-value of the estimated edge between gene j and h is approximated by the quantile of $\tilde {r}_{jh} $ in the null distribution *F*^null^ with Benjamini-Hochberg adjustment [19] to avoid multiple comparison problems.

$$D\xrightarrow{\text{permutate}} \left\{ \begin{array}{ccc} D^{(1)} & \xrightarrow{\text{ENA}} & \tilde{R}^{(1)}\\ \vdots & & \vdots \\ D^{(M)} & \xrightarrow{\text{ENA}} & \tilde{R}^{(M)} \end{array} \right\} \rightarrow F^{\text{null}}, $$

$${\begin{aligned} {p}-value(jh)=BHadjust\left(\frac{\#\text{ of }\tilde{r}_{jh}\leq \text{ permutated}~r \text{ value in }F^{\text{null}}} {\text{Total }\#\text{ of }\tilde{r}_{jh}\leq \text{ permutated}~r \text{ value in }F^{\text{null}}}\right). \end{aligned}} $$

In the simulation studies, we ensembled the networks constructed by NS, GLASSO, GLASSO-SF, PCACMI, SPACE, and BayesianGLASSO. GeneNet and CMI2NI were excluded because GeneNet performed the worst in all the scenarios (Additional file 4: Figure S1-S8) and CMI2NI produced the exact same results as PCACMI in default settings. We run all the processes in a single node of UT Southwestern BioHPC cluster (Intel(R) Xeon(R) CPU E5-2650 v3 @ 2.30GHz, 32GB RAM).

## Results

**Fig. 2:**
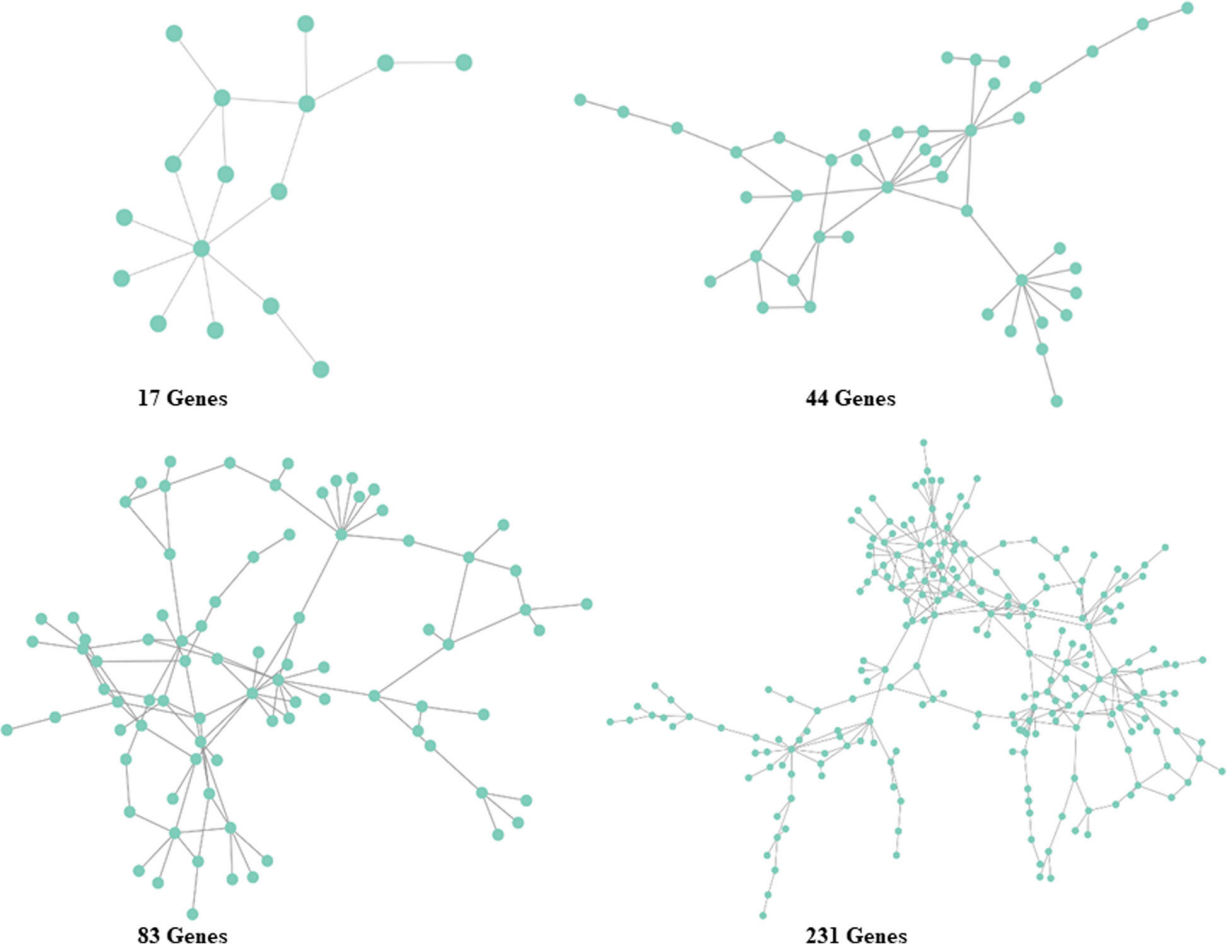
The four real protein-protein interaction networks used in the simulation study

To comprehensively evaulate different models, we simulated co-expression data from four real protein-protein interaction networks (Fig. 2) used in Allen et al. [5], which was selected Keshava Prasad et al. [20]. See the download link for the four real network structure in the Availability of data and materials section. Details of the generative model are discussed below. We investigated the performance of each method for data with various noise levels and sample sizes.

### Generative model

We used Gaussian graphical models that are mainly used to infer the gene association network to simulate expression data. Let **y**_*i*_=(*y*_*i*1_,…,*y*_*ij*_,…,*y*_*ip*_) denotes the collection of expression levels for each gene observed in sample *i*. This was simulated from a zero-mean multivariate normal distribution *y*_*i*_=MN(**0**_*p*_, ***Σ***+*ε*^2^**I**_*p*×*p*_), where **0**_*p*_ denotes the *p*-dimension zero vector and **I**_*p*×*p*_ denotes the *p*-by-*p* identity matrix. For the covariance matrix ***Σ***, we generated its concentration matrix ***Ω***=***Σ***^−1^ following Peng, et al. [13]. The initial matrix ***Ω*** was created by setting

$${\begin{aligned} \omega_{jh}=\left\{ \begin{array}{ll} 1 &, \ j = h \\ 0 &, \ j \neq h, j \nsim h \\ 0.5\text{Uniform}(-1,-0.5)+0.5\text{Uniform}(0.5,1) &,\ j \neq h,j \sim h \end{array} \right., \end{aligned}} $$

where *Uniform*(*a*,*b*) represents uniform distribution on interval (*a*,*b*), *j*∼*h* indicates that there is an edge between gene *j* and *h*, $j\nsim h $ means otherwise. The network structure was chosen from one of the four real protein-protein interaction networks [20, 21], each of which was approximately scale-free (see Fig. 2). Then, the non-zero elements in ***Ω*** were rescaled to assure positive definiteness. Specifically, for each row, we first summed the absolute values of the off-diagonal elements, and divided each off-diagonal entry by 1.5-fold their sum. Next, we averaged this rescaled matrix with its transpose to ensure symmetry. We then set 0.1 to those non-zero entries with absolute value smaller than 0.1. After that, the inverse of the final matrix was denoted by **A**=***Ω***^−1^. Each element in the covariance matrix ***Σ*** was determined by $\delta _{jh}=\alpha _{jh}/\sqrt {\alpha _{jj}\alpha _{hh}}$. For the noise level *ε*, we considered three cases: *ε*=0, 0.1, 0.5.

### Performance metric

We evaluated the result of each method by plotting its operating characteristic curve (ROC) and calculating the area under the ROC curve (AUC). As different methods generate different outputs, we used their corresponding approaches to plot ROC curves for a fair comparison. GeneNet and BayesianGLASSO yield a continuous estimate of each partial correlation *ρ*_*jh*_. They do not require a tuning parameter. Thus, an edge between gene *j* and *h* was determined if the absolute value of *ρ*_*jh*_ was greater than a certain threshold. Then the ROC curves were obtained by plotting false positive rates (FPRs) against true positive rates (TPRs) under different thresholds. For mutual information-based methods, we choose the tuning parameter *α*=0.03 as suggested by the authors [17, 18]. Then, an edge between gene *j* and *h* was determined if the estimated entropy was greater than a threshold. The ROC curves were obtained by plotting FPRs against TPRs under different thresholds. Note that we only included PCACMI in the simulation, since CMI2NI produced the same result as PCACMI did. For the other methods that need a tuning parameter, the ROC curves were obtained by plotting FPRs and TPRs under different choices of the tuning parameter.

### Result summarization

As shown in the result of simulation study (Additional file 4: Figure S1-S8), BayesianGLASSO and ENA generally outperform other methods, which is consistent with the literature [6, 16]. Besides, mutual information-based methods also show competitive results. NS, GLASSO, and GLASSO-SF, which share the same strategy, have similar accuracy. As the earliest developed method, GeneNet has significantly lagged performance. Not surprisingly, all methods lose power when either a higher level of noise manifests or a smaller number of samples is generated.

We also logged the computational time of each method in Table S2 (Additional file 5). The Bayesian method consumed several orders of magnitude more time, and it soon went beyond real applicability when the number of genes in the network increased to hundreds. Most other methods shared similar efficacy in the simulation settings, with mutual information-based methods being a little slower.

## Discussion

GeNeCK infers a gene-gene connection based on the expression pattern of the two genes. It can provide a hint of their potential functional relationship, but does not necessarily imply a real biological interaction. One should be very cautious when interpreting the result, especially when the tuning parameter is out of a reasonable range (e.g. an almost fully connected network may be a sign of choosing a problematic parameter value). As different methods use different measurements to evaluate the confidence of estimated edges (e.g. partial correlation, mutual information), this may not be easy to interpret for users with little statistical background. We suggest users choose the ENA method, which outputs *p*-values to indicate the significance of gene-gene connections. More importantly, it generally achieves the best performance. For extended methods (EGLASSO and ESPACE) that allow for the “hub genes” specification, additional attention needs to be paid when choosing the value for the confidence index *α*. The *α* value can be selected by different statistical methods, such as the generalized information criterion (GIC) [22]. In practice, we suggest an initial try with no or a very weak prior brief to see if the genes of interest are picked up by the algorithm. Usually a very small *α* value is not desired, as the influence of hub genes should already be presented in the data if the prior information is correct. Otherwise this can lead to a biased result.

## Conclusion

Reconstructions of gene networks from gene expression data greatly facilitate our understanding of underlying biological mechanisms and provide new opportunities for drug and biomarker discoveries. GeNeCK, the online tool kit presented in this paper, enables us to integrate various statistical methods to construct gene networks based on gene expression data. Furthermore, the information of hub genes, which usually play an essential role in gene regulation and biological processes, could be incorporated into GeNeCK to improve the performance of the related methods. It is believed that the tool will cater to a wide audience in the field of biology.

## Availability and requirements

**Project name:** GeNeCK

**Project home page:** http://lce.biohpc.swmed.edu/geneck/

**Operating systems:** Windows, Linux and Mac

**Programming language:** PHP, HTML, JavaScript and R

**License:** GPL

## Additional files

Additional file 1 **Figure S9.** GeNeCK user guide. A simple tutorial on how to run GeNeCK. (DOCX 195 kb)

Additional file 2 **Figure S10.** External visulization of GeNeCK inference result. Example of how to import GeNeCK output to Cytoscape for enhanced visulization. (DOCX 326 kb)

Additional file 3 **Table S1.** Summary of basic information of different methods in GeNeCK. (DOCX 14 kb)

Additional file 4 **Figure S1-S8.** Comparison of model performance of different methods in simulation studies. Network structures are based on real protein-protein interaction networks. Expression data are simulated under different noise levels. (DOCX 776 kb)

Additional file 5 **Table S2.** Summary of runtime of different methods in GeNeCK. (DOCX 18 kb)

## Abbreviations

AUC: Area under curve
BayesianGLASSO: Bayesian graphical LASSO
CMI2NI: Conditional mutual inclusive information-based network inference
EGLASSO: Extended GLASSO
ENA: Ensemble-based network aggregation
ESPACE: Extended SPACE
GeNeCK: Gene network construction tool kit
GLASSO: Graphical LASSO
GLASSO-SF: GLASSO with reweighted strategy for scale-free network
GRN: Gene regulatory network
MI: Mutual information
NS: Neighborhood selection
PCACMI: Path consistency algorithm based on conditional mutual information
ROC: Operating characteristic curve
SPACE: Sparse partial correlation estimation
