# Supplementary material for: GeNeCK: a web server for gene network construction and visualization
Source: BMC Bioinformatics. 2019 Jan 7;20:12. doi: 10.1186/s12859-018-2560-0 (PMC6323745; doi:10.1186/s12859-018-2560-0)
Supplement: Supplementary file 1 — Figure S9. GeNeCK user guide. A simple tutorial on how to run GeNeCK. (DOCX 195 kb) [file 12859_2018_2560_MOESM1_ESM.docx]

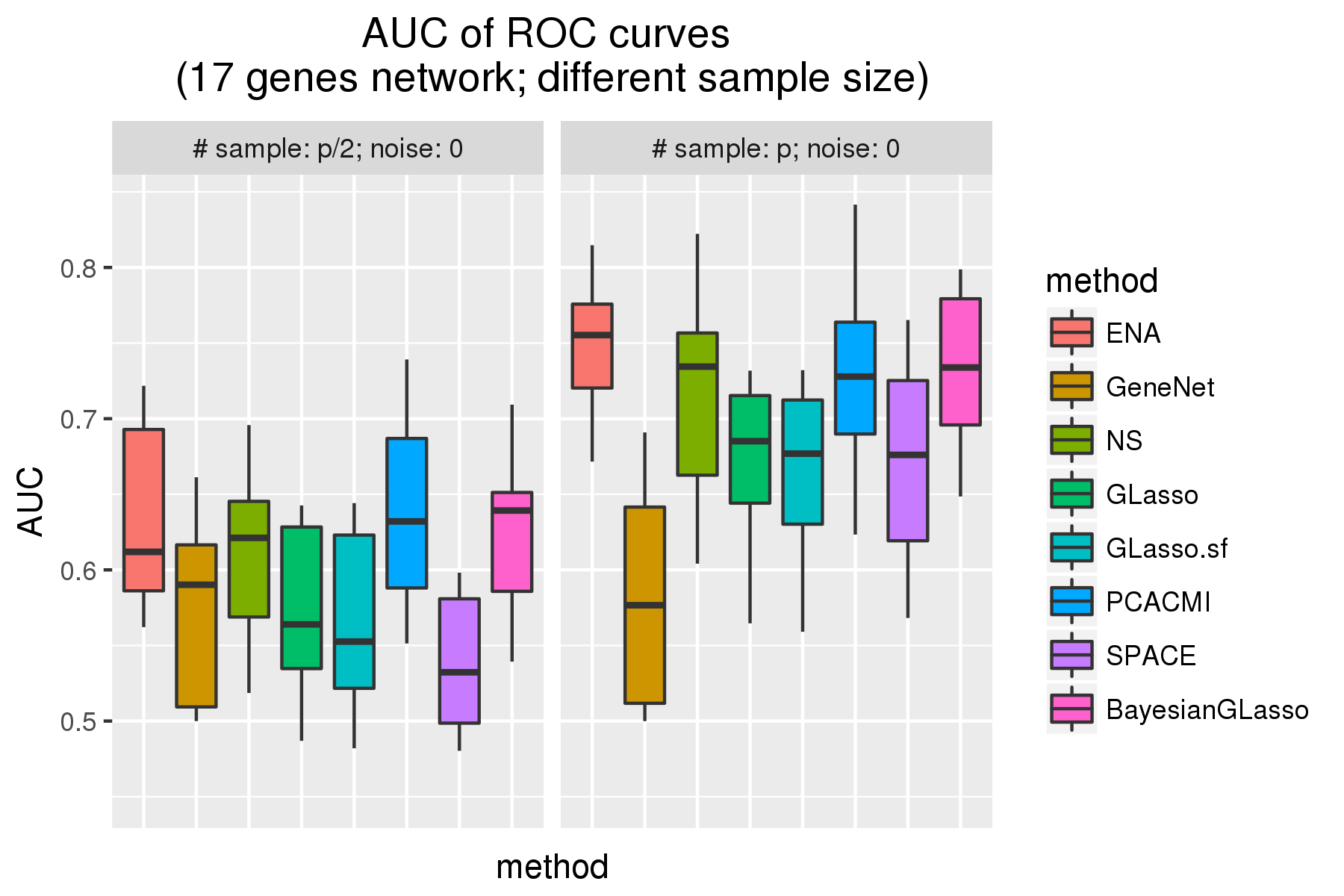


**Figure S1.** Comparison of model performance across different sample sizes in the 17 genes network.


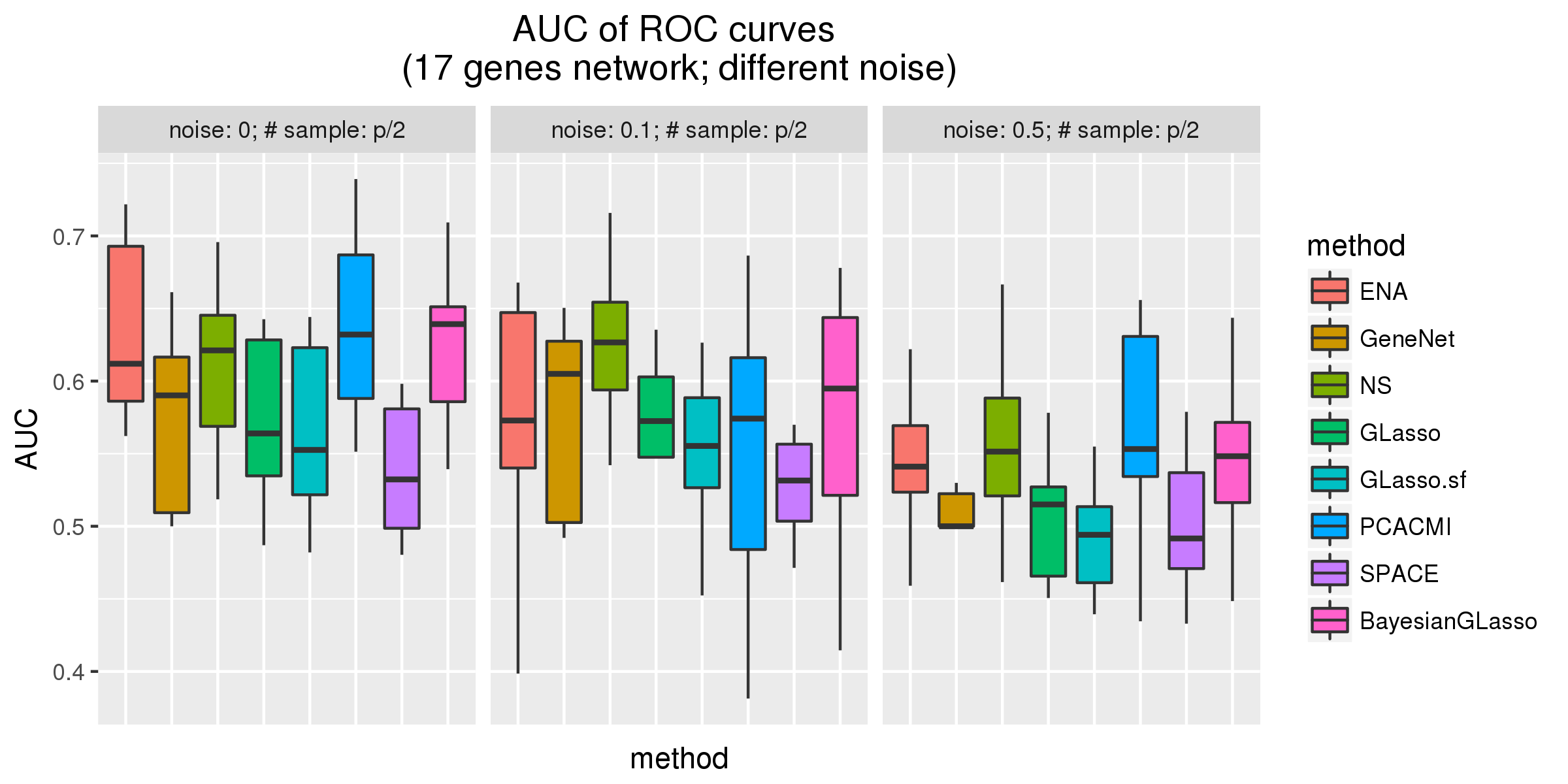


**Figure S2.** Comparison of model performance across different noise levels in the 17 genes network.

**
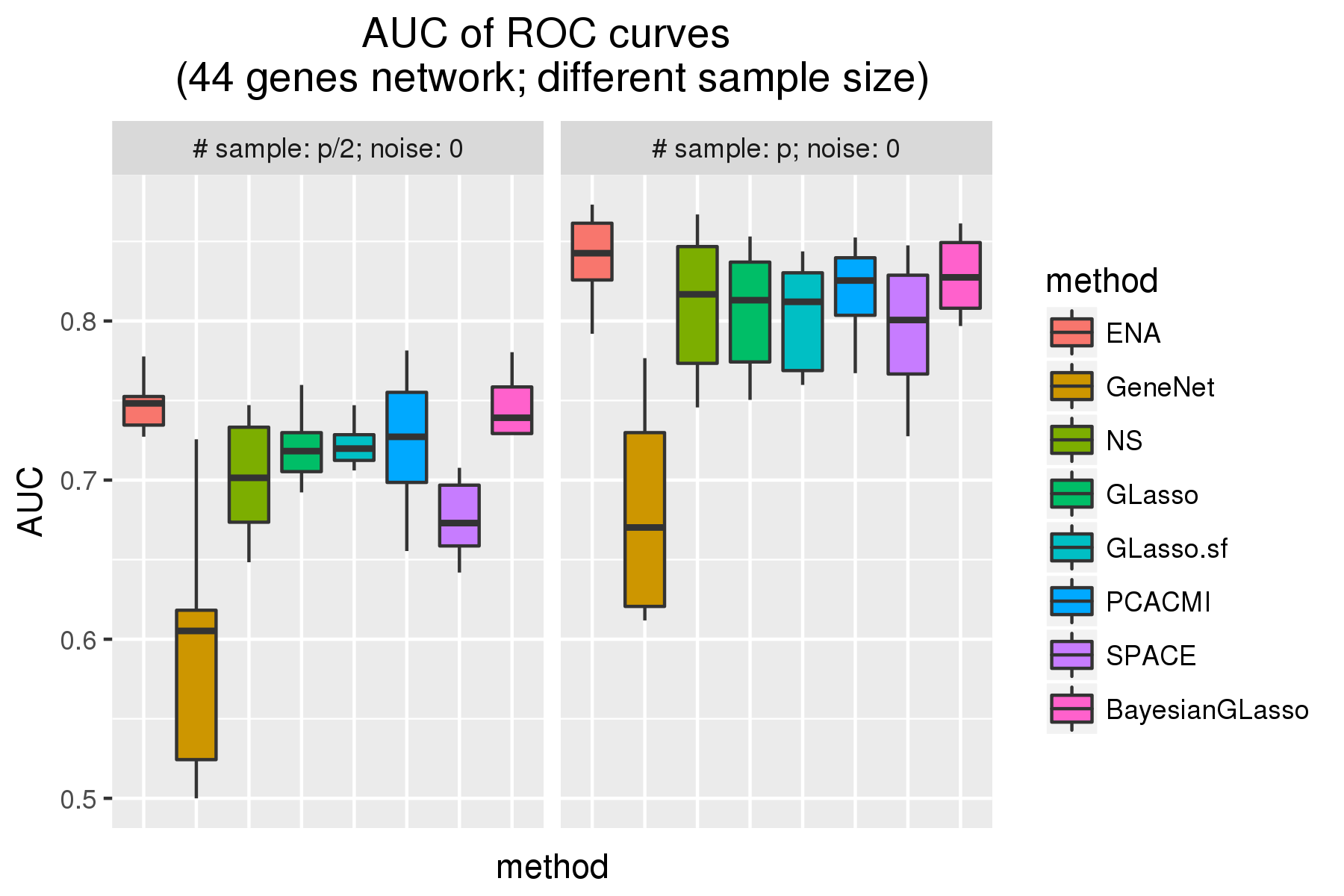
**

**Figure S3.** Comparison of model performance across different sample sizes in the 44 genes network.

**
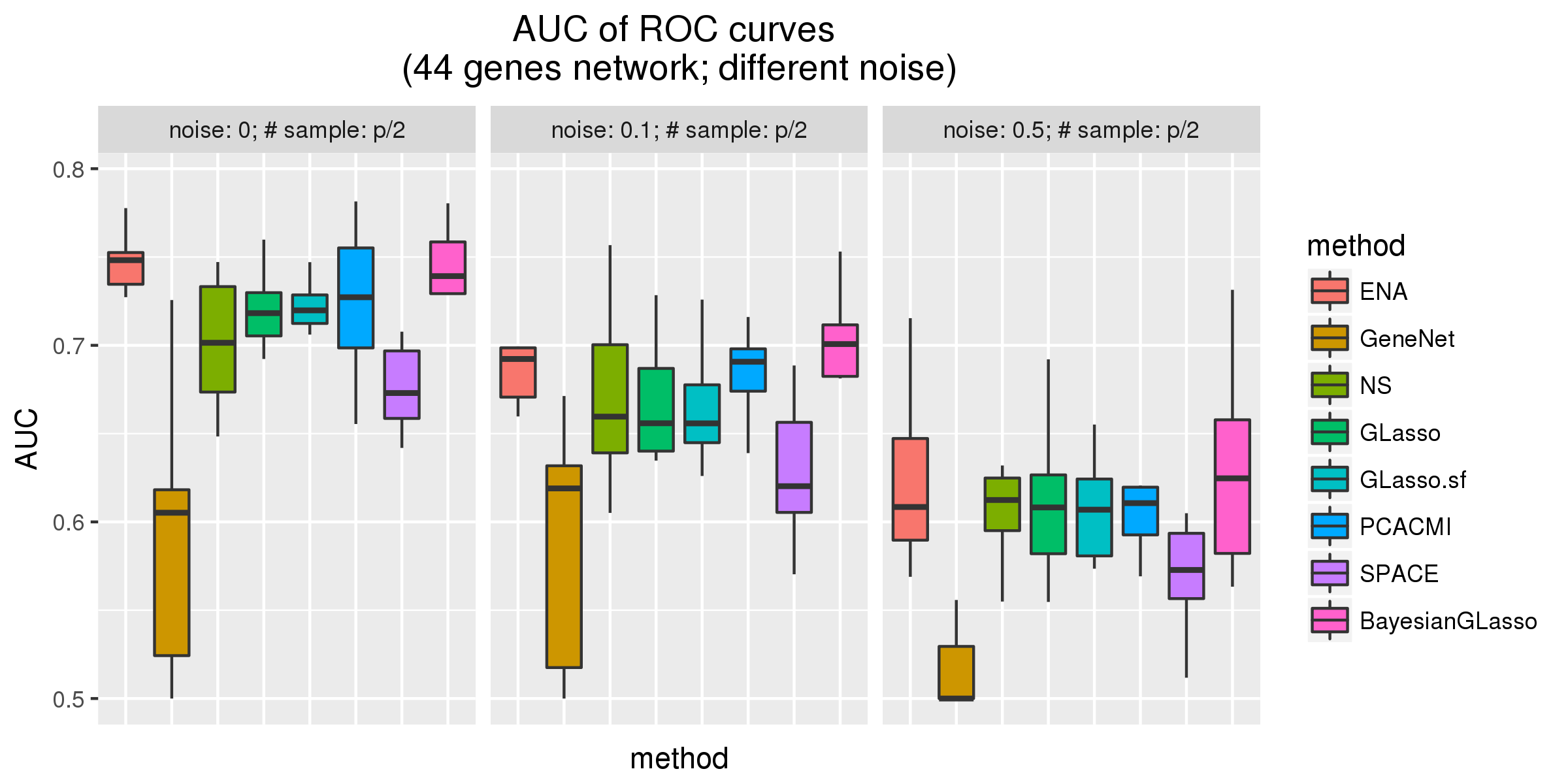
**

**Figure S4.** Comparison of model performance across different noise levels in the 44 genes network.

**
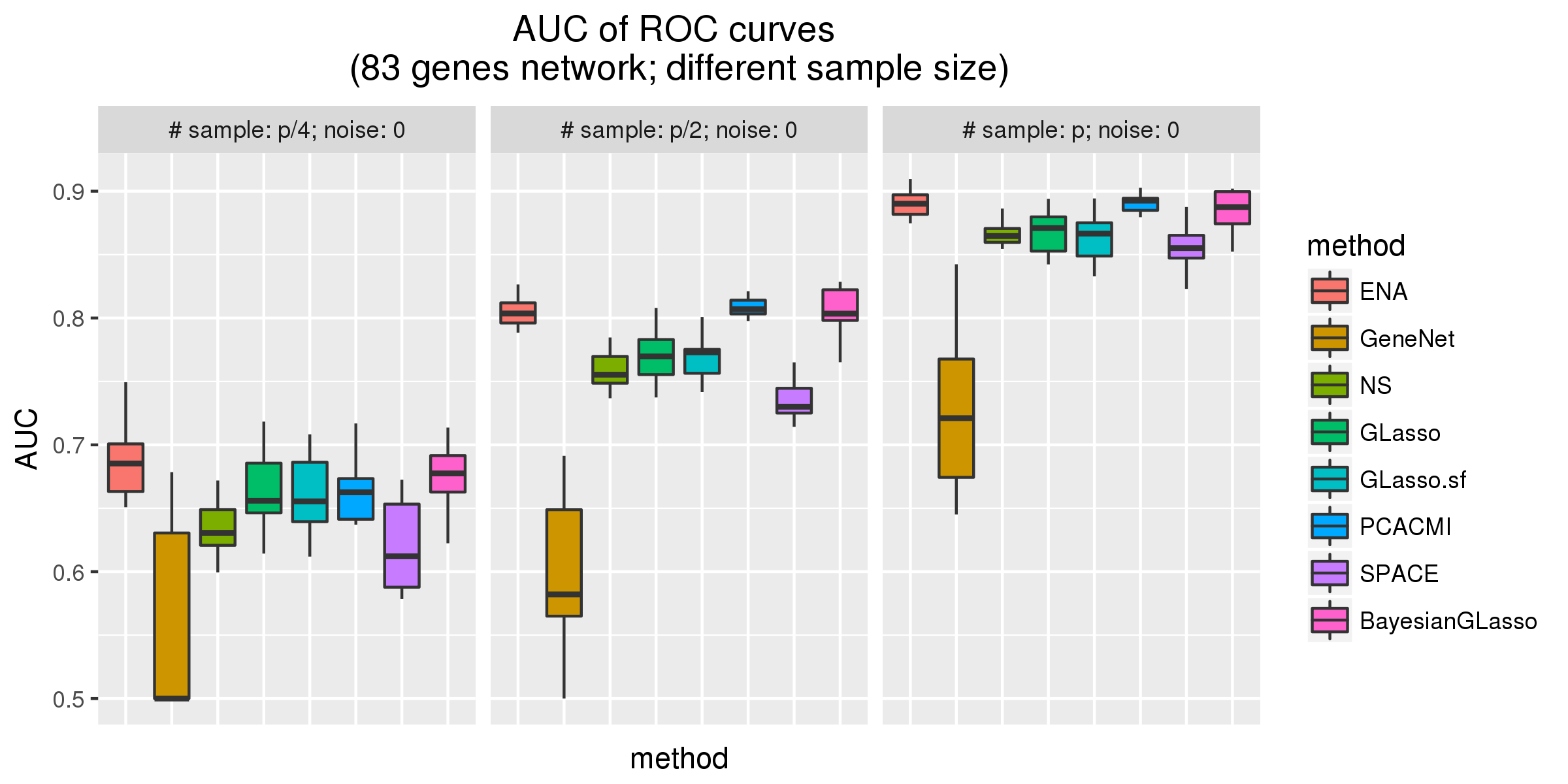
**

**Figure S5.** Comparison of model performance across different sample sizes in the 83 genes network.

**
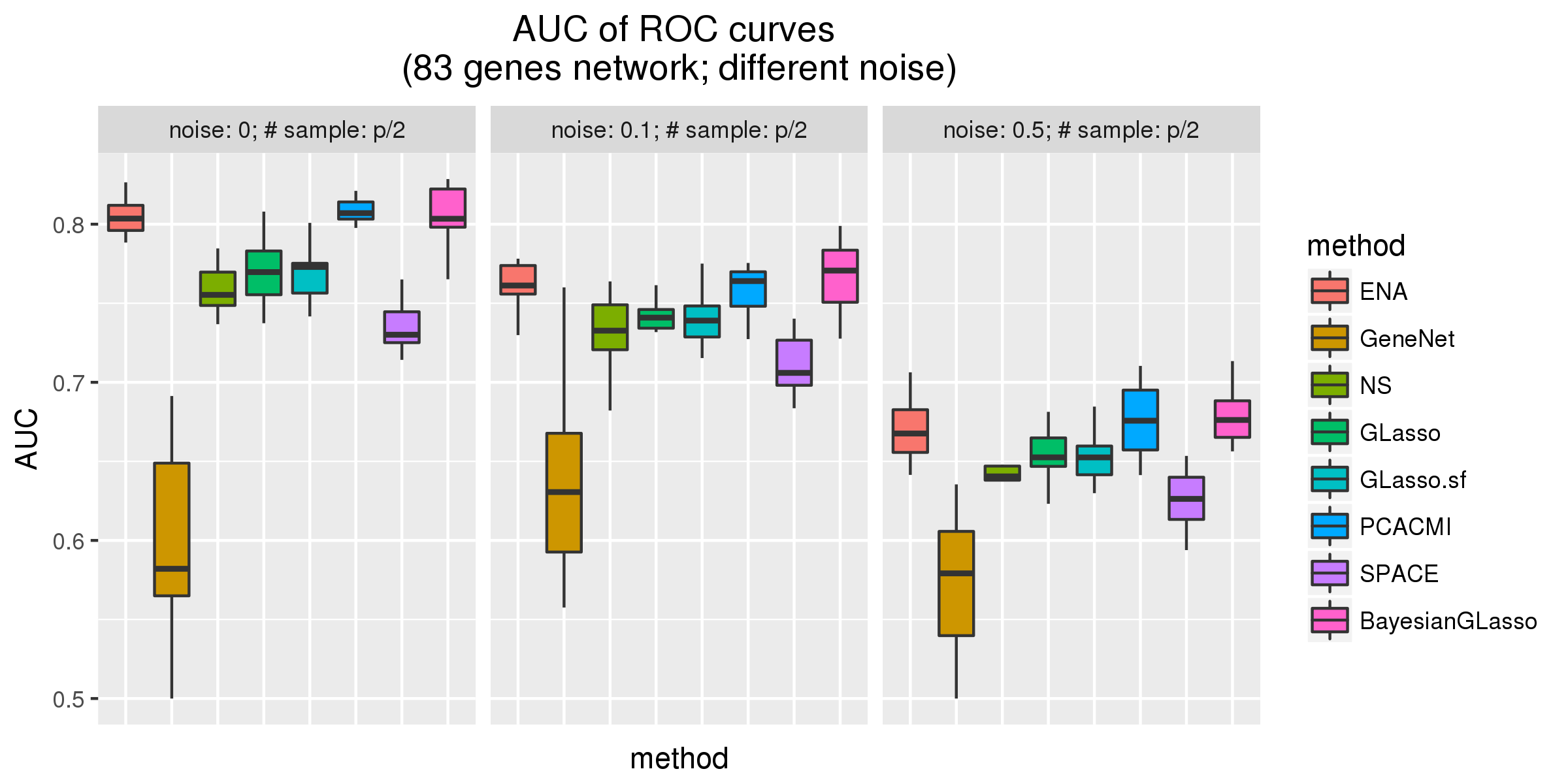
**

**Figure S6.** Comparison of model performance across different noise levels in the 83 genes network.

**
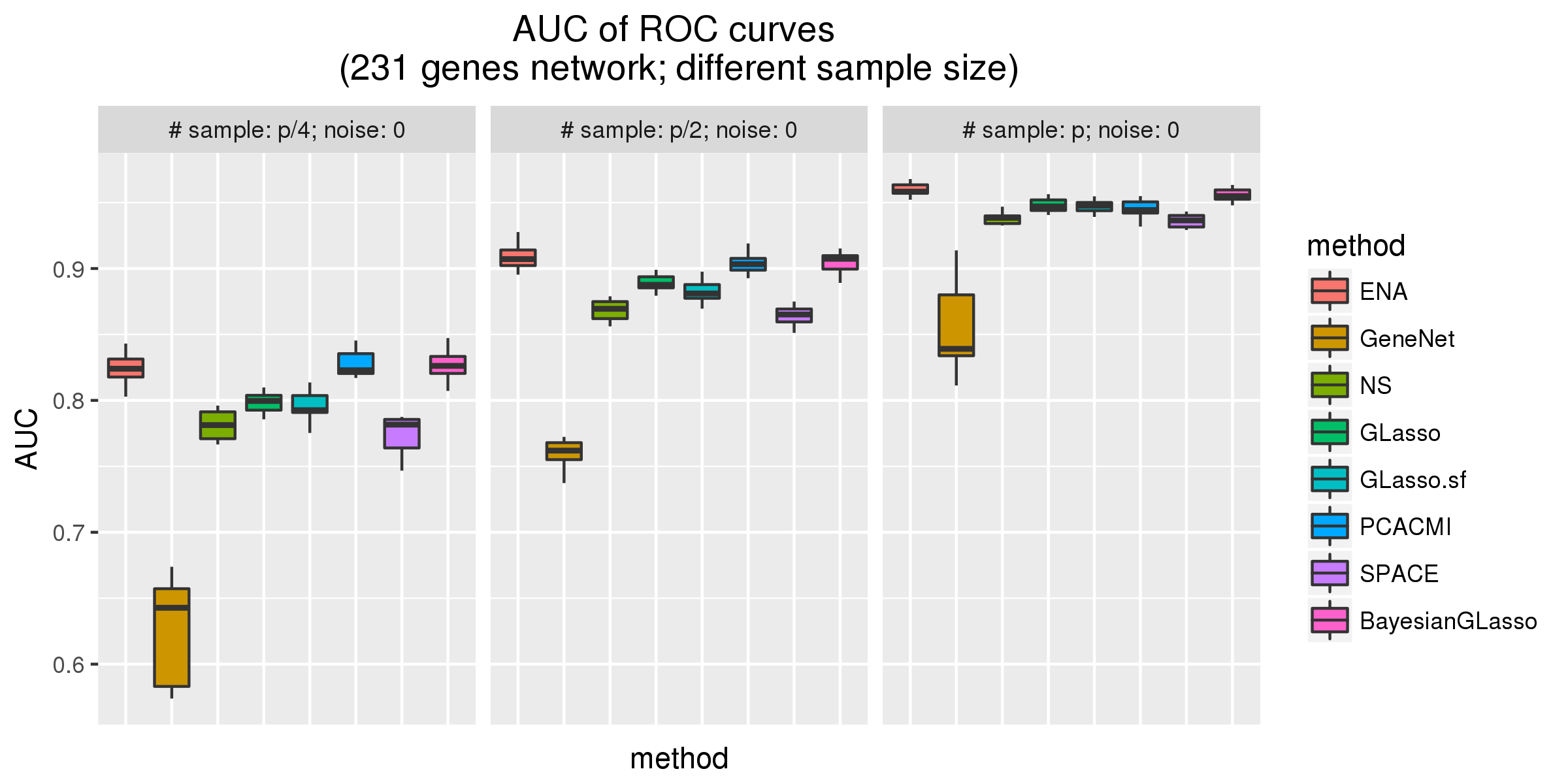
**

**Figure S7.** Comparison of model performance across different sample sizes in the 231 genes network.

**
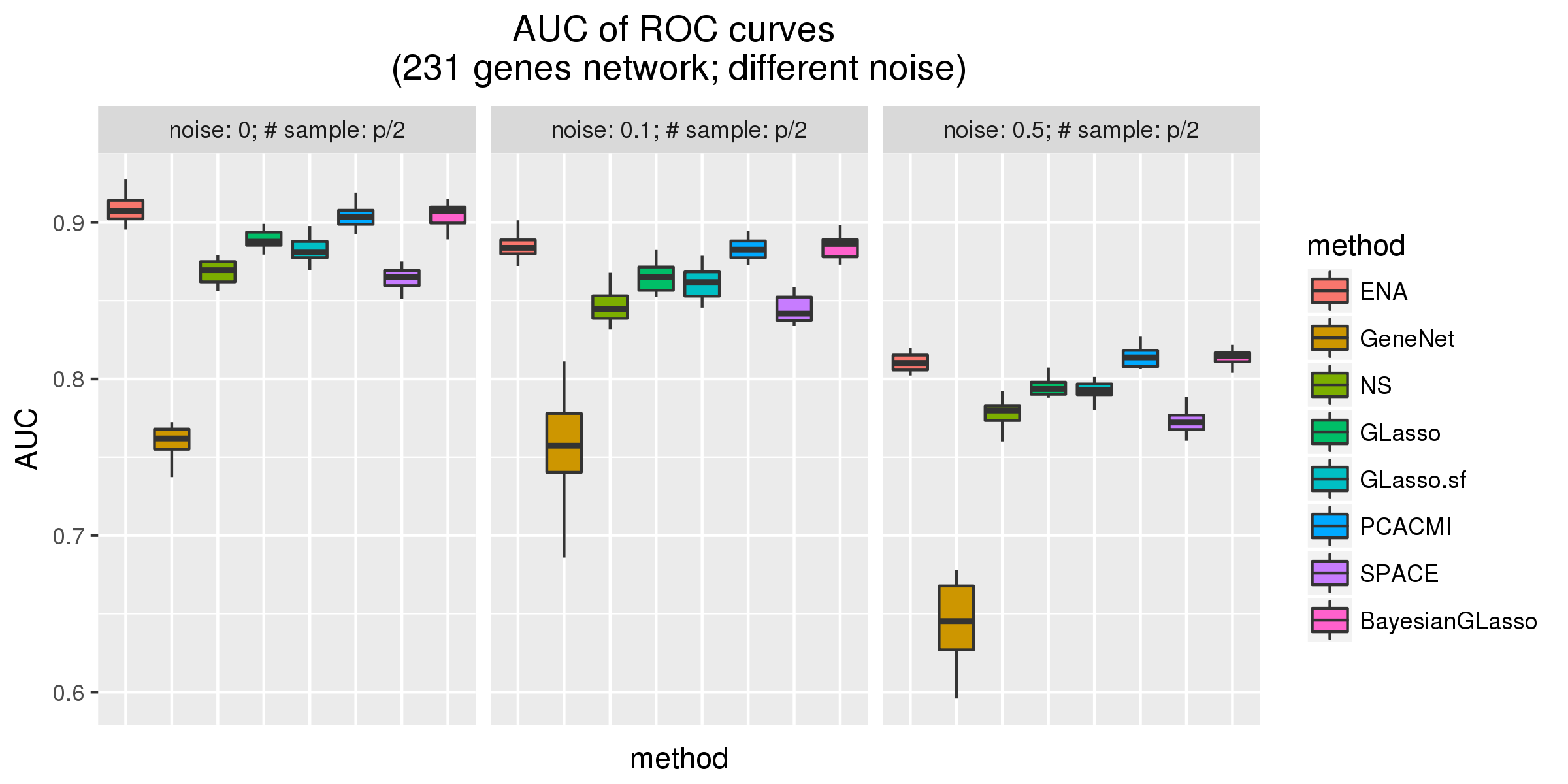
**

**Figure S8.** Comparison of model performance across different noise levels in the 231 genes network.
