# Supplementary figures and images for: GeNeCK: a web server for gene network construction and visualization
Source: BMC Bioinformatics. 2019 Jan 7;20:12. doi: 10.1186/s12859-018-2560-0 (PMC6323745; doi:10.1186/s12859-018-2560-0)

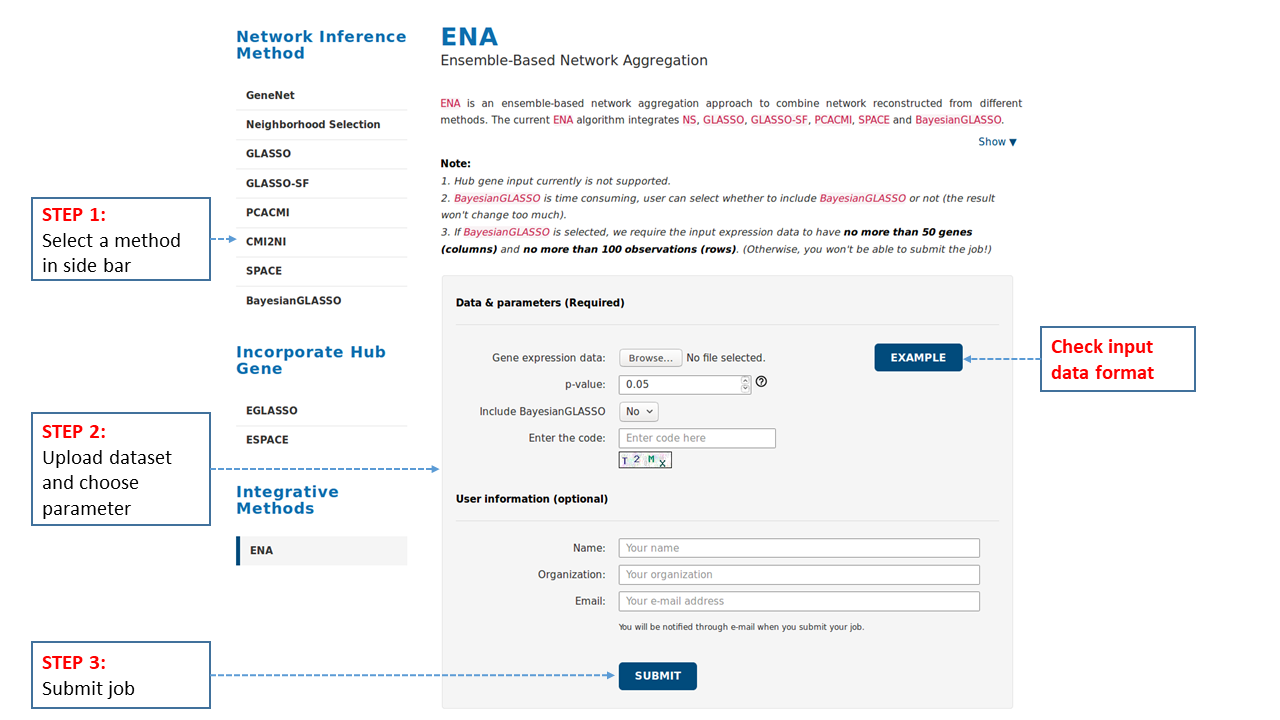
**Figure S9.** GeNeCK user guide.

Supplement: Supplementary file 2 — Figure S10. External visulization of GeNeCK inference result. Example of how to import GeNeCK output to Cytoscape for enhanced visulization. (DOCX 326 kb) [file 12859_2018_2560_MOESM2_ESM.docx]
