# Supplementary material for: GeNeCK: a web server for gene network construction and visualization
Source: BMC Bioinformatics. 2019 Jan 7;20:12. doi: 10.1186/s12859-018-2560-0 (PMC6323745; doi:10.1186/s12859-018-2560-0)
Supplement: Supplementary file 3 — Table S1. Summary of basic information of different methods in GeNeCK. (DOCX 14 kb) [file 12859_2018_2560_MOESM3_ESM.docx]

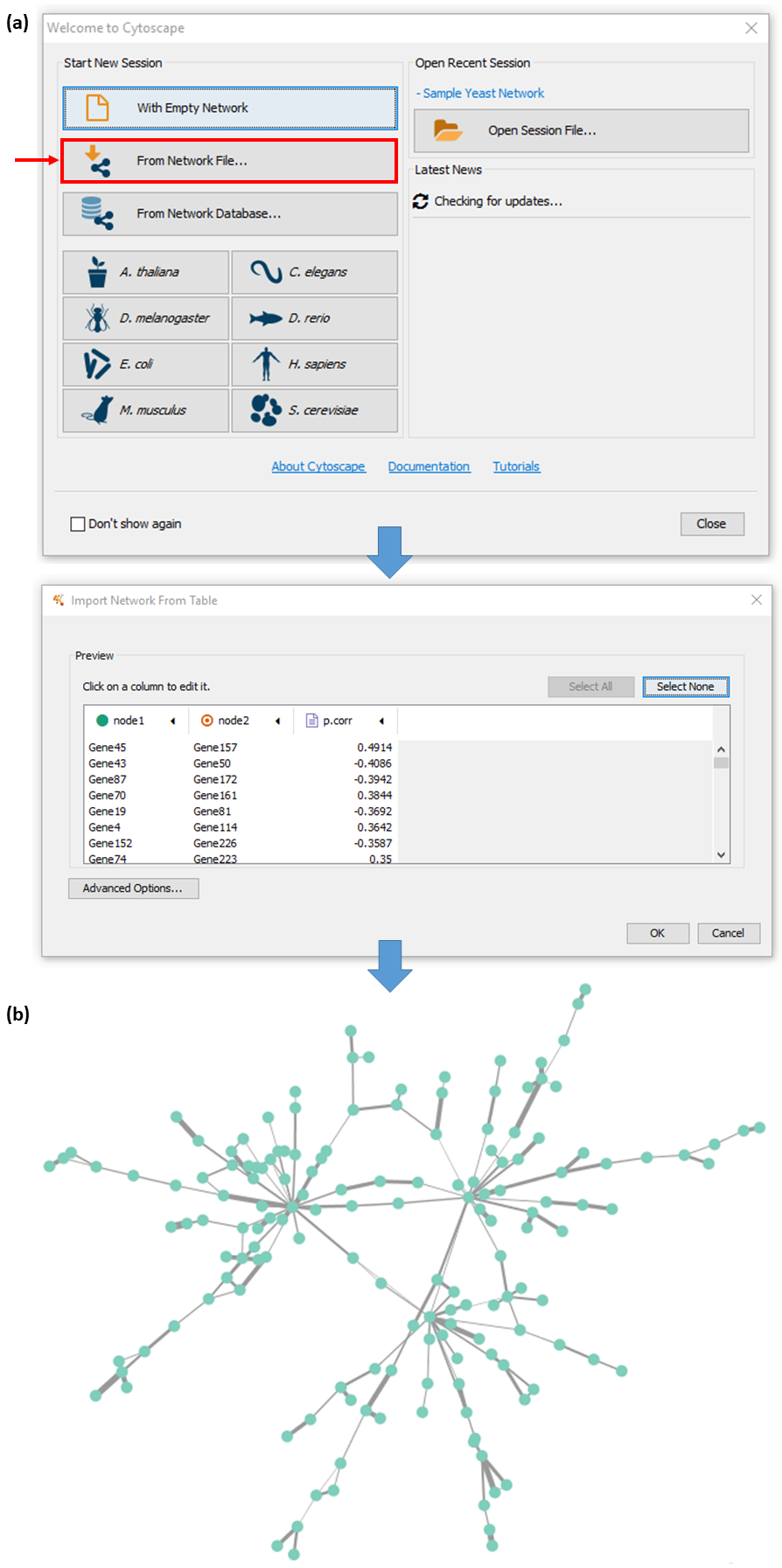


**Figure S10.** (a) Import GeNeCK inferred network file to Cytoscape. (b) Network visulization in Cytoscape. Edge widths are mapped to estimated connection strength. Thick edges represent connections with high confidence.
