# Supplementary material for: GeNeCK: a web server for gene network construction and visualization
Source: BMC Bioinformatics. 2019 Jan 7;20:12. doi: 10.1186/s12859-018-2560-0 (PMC6323745; doi:10.1186/s12859-018-2560-0)
Supplement: Supplementary file 4 — Figure S1-S8. Comparison of model performance of different methods in simulation studies. Network structures are based on real protein-protein interaction networks. Expression data are simulated under different noise levels. (DOCX 776 kb) [file 12859_2018_2560_MOESM4_ESM.docx]

**Table S1.** Summary of basic information of different methods in GeNeCK

| Method | Category | Hub info. | Implementation | Package | Reference |
| --- | --- | --- | --- | --- | --- |
| GeneNet | Partial correlation | No | R | GeneNet | Schafer et al. [11] |
| NS | Partial correlation | No | R | CDLasso | Meinshausen et al. [12] |
| GLASSO | Likelihood | No^*^ | R | glasso | Friedman et al. [14] |
| GLASSO-SF | Likelihood | No^*^ | R | glasso | Liu et al. [15] |
| PCACMI | Mutual information | No | MATLAB^†^ | pca_cmi.m | Zhang et al. [17] |
| CMI2NI | Mutual information | No | MATLAB^†^ | CMI2NI.m | Zhang et al. [18] |
| SPACE | Partial correlation | No | R | space | Peng et al. [13] |
| BayesianGLASSO | Likelihood | No^*^ | R | BayesianGLasso | Wang et al. [16] |
| EGLASSO | Likelihood | Yes | R | N/A | Yu et al. [7] |
| ESPACE | Partial correlation | Yes | R | espace | Yu et al. [7] |
| ENA | Ensemble | No | R | ENA | Zhong et al. [6] |

^*^ These methods might be adapted to incorporate hub information.
