# Supplementary material for: GeNeCK: a web server for gene network construction and visualization
Source: BMC Bioinformatics. 2019 Jan 7;20:12. doi: 10.1186/s12859-018-2560-0 (PMC6323745; doi:10.1186/s12859-018-2560-0)
Supplement: Supplementary file 5 — Table S2. Summary of runtime of different methods in GeNeCK. (DOCX 18 kb) [file 12859_2018_2560_MOESM5_ESM.docx]

**Table S2.** Summary of runtime of different methods in GeNeCK

| *n* | *p* | $\boldsymbol{\varepsilon}$ | GeneNet | NS | GLasso | GLasso-SF | PCACMI | SPACE | BayesianGLasso | ENA^*^ |
| --- | --- | --- | --- | --- | --- | --- | --- | --- | --- | --- |
| 17 | 9 | 0.0 | 2.011 | 0.002 | 0.002 | 0.008 | 1.548 | 0.006 | 31.331 | 0.010 |
| 17 | 9 | 0.1 | 0.254 | 0.003 | 0.003 | 0.011 | 1.740 | 0.009 | 31.228 | 0.010 |
| 17 | 9 | 0.5 | 0.823 | 0.002 | 0.004 | 0.011 | 1.732 | 0.010 | 33.844 | 0.011 |
| 17 | 17 | 0.0 | 1.414 | 0.003 | 0.002 | 0.008 | 1.213 | 0.007 | 35.438 | 0.012 |
| 44 | 22 | 0.0 | 0.118 | 0.007 | 0.013 | 0.073 | 16.362 | 0.027 | 197.855 | 0.027 |
| 44 | 22 | 0.1 | 0.153 | 0.011 | 0.021 | 0.115 | 9.824 | 0.041 | 181.091 | 0.026 |
| 44 | 22 | 0.5 | 0.195 | 0.009 | 0.020 | 0.117 | 3.359 | 0.049 | 196.195 | 0.027 |
| 44 | 44 | 0.0 | 0.161 | 0.013 | 0.014 | 0.072 | 2.207 | 0.040 | 168.841 | 0.025 |
| 83 | 20 | 0.0 | 0.192 | 0.020 | 0.088 | 0.605 | 1471.080 | 0.093 | 6376.053 | 0.109 |
| 83 | 42 | 0.0 | 0.261 | 0.035 | 0.063 | 0.384 | 2.465 | 0.086 | 3727.393 | 0.129 |
| 83 | 42 | 0.1 | 0.201 | 0.040 | 0.074 | 0.513 | 2.431 | 0.102 | 4317.689 | 0.153 |
| 83 | 42 | 0.5 | 0.255 | 0.036 | 0.079 | 0.547 | 1.987 | 0.138 | 7227.523 | 0.116 |
| 83 | 83 | 0.0 | 0.165 | 0.042 | 0.039 | 0.196 | 1.559 | 0.081 | 3420.564 | 0.115 |
| 231 | 58 | 0.0 | 0.771 | 0.273 | 1.007 | 8.302 | 8.670 | 0.790 | ≈ 1 week | 1.396 |
| 231 | 116 | 0.0 | 16.771 | 0.505 | 3.159 | 3.890 | 5.602 | 0.641 | ≈ 1 week | 1.295 |
| 231 | 116 | 0.1 | 0.888 | 0.421 | 0.635 | 3.551 | 4.984 | 0.717 | ≈ 1 week | 1.410 |
| 231 | 116 | 0.5 | 0.910 | 0.510 | 0.898 | 6.208 | 5.153 | 1.228 | ≈ 1 week | 1.443 |
| 231 | 231 | 0.0 | 0.985 | 0.656 | 0.458 | 1.993 | 5.180 | 1.210 | ≈ 1 week | 1.557 |

^*^ The computational time of ENA was measured by its aggregation step. The time for construction of network to ensemble was not considered.
